# First establishment of population-specific brain volume reference standards for Chinese children and adolescents: an AI-based segmentation study

**DOI:** 10.3389/fped.2025.1723455

**Published:** 2025-12-12

**Authors:** Hangchen Qian, Jingyun Liao, Jihao Cheng

**Affiliations:** 1 Department of Radiology, The Second Affiliated Hospital of Zhejiang Chinese Medical University, Hangzhou, China; 2 Department of Radiology, Affiliated Xiaoshan Hospital of Hangzhou Normal University, Hangzhou, China

**Keywords:** automated brain segmentation, brain structure volume, magnetic resonance imaging (MRI), children and adolescents, developmental neuroscience

## Abstract

**Objective:**

To establish the first AI-based, population-specific reference standards for brain structural volumes in healthy Chinese children and adolescents, and to elucidate unique developmental trajectories that may differ from existing Western norms.

**Methods:**

In this cross-sectional study, 1,100 healthy participants aged 6–18 years were enrolled. T1-weighted images were acquired using a 3.0T MRI scanner, and regional brain volumes were quantified using an AI-based automated segmentation tool. Multiple comparisons were corrected using the false discovery rate (FDR) method. Differences in brain volume by sex, age, and hemisphere, as well as correlations with age, were analyzed.

**Results:**

After controlling for age, males exhibited larger total intracranial volume (*P* < 0.001) and left cerebral white matter volume (*P* = 0.015), whereas females showed larger volumes in the bilateral parahippocampal gyri (left: *P* = 0.023; right: *P* = 0.011) and left fusiform gyrus (*P* = 0.026). Significant hemispheric asymmetries were observed in multiple regions. Cerebrospinal fluid (CSF) and bilateral white matter volumes were positively correlated with age (*P* < 0.001), while volumes in certain frontal and temporal lobe regions, as well as subcortical structures, were negatively correlated with age (*P* < 0.05).

**Conclusion:**

This study is the first to establish reference values for brain volume in healthy Chinese children and adolescents aged 6–18 years using AI-based automated segmentation. It reveals sex- and age-related differences in brain structural development, thereby providing a valuable quantitative reference for future research on brain development deviations. These population-specific standards may provide a theoretical foundation for improving diagnostic accuracy in Chinese children, though their actual utility in reducing misclassification requires future empirical validation.

## 1 Introduction

The human brain comprises multiple functional regions involved in physiological processes such as memory storage, language processing, and motor control (1). In neurological disorder research, morphological indicators such as volume changes in specific brain regions hold significant clinical value. MRI renowned for its excellent soft-tissue contrast and detailed anatomical visualization, has become a pivotal tool in brain research. Advanced AI-based brain segmentation techniques, which utilize deep neural networks to process MRI data, can process vast amounts of imaging data to accurately quantify three-dimensional volumetric parameters of brain substructures, enabling the detection of subtle changes in brain tissue (2). Compared to widely used tools like FreeSurfer and AFNI, the uAI-Brain segmentation system employed in this study offers significant improvements in computational efficiency (e.g., processing times reduced by ∼30% in validation studies) and achieves high accuracy (Dice coefficients of 0.85–0.95 for major brain regions), as validated against manual segmentation in our Methods section.

Currently, mainstream international reference standards for brain volume are primarily based on Western populations. Their racial and geographical specificity may introduce bias when applied to Chinese pediatric populations (3). Although recent studies have begun to focus on brain development characteristics in Asian populations (4), existing domestic research has largely concentrated on neurodegenerative and psychiatric disorders such as Alzheimer's disease and schizophrenia in middle-aged and older adults (5, 6). Large-scale systematic studies employing AI-based automated segmentation technology to examine brain structural development in healthy school-aged children and adolescents in China are still scarce. Therefore, there is a critical unmet need for a large-scale, AI-driven, and culturally specific brain volume atlas for the developing Chinese population.

This study aims to fill this gap by establishing the first population-specific reference standards for brain structural volumes in Chinese children and adolescents using an AI-based automated segmentation tool. The establishment of these Chinese-specific norms is a critical first step, providing the necessary baseline data against which future studies can rigorously test for population-specific variations in neurodevelopmental trajectories compared to Western cohorts. To this end, we pioneer the use of an AI-based automated segmentation tool to construct a standardized reference database of brain volumes for a cohort of 1,100 healthy Chinese children and adolescents aged 6–18 years, addressing a critical gap in this field. The established reference values provide a foundational tool and population-specific benchmark. In a future clinical setting, these standards could allow radiologists to quantify individual brain development against a Chinese-specific norm, potentially reducing reliance on subjective visual assessment and Western-derived standards. This approach could potentially enhance the objectivity of early diagnosis for developmental disorders, subject to future validation.

## 2 Materials and methods

### 2.1 Subjects

A total of 1,100 healthy child and adolescent volunteers, scanned between January 2017 and July 2024, were included in this study. The cohort comprised 495 males and 555 females, aged 6–18 years (mean age: 12.43 ± 3.67 years). Participants were divided into four age groups (A-D): 6–9, 10–12, 13–15, and 16–18 years, with 255, 270, 270, and 255 subjects in each group, respectively. Participants were divided into four age groups (A–D): 6–9, 10–12, 13–15, and 16–18 years, with 255, 270, 270, and 255 subjects in each group, respectively. This grouping was based on key neurodevelopmental stages: early school age (6–9 years), pre-adolescence (10–12 years), early adolescence (13–15 years), and late adolescence (16–18 years), which align with established developmental trajectories in brain maturation (1, 3).

#### 2.1.1 Inclusion criteria

① No history of psychiatric disorders or relevant family history; ② No history of head trauma or organic brain lesions; ③ No use of psychotropic medications within the past 6 months (defined as “recent”), as psychotropic drugs may have prolonged effects on brain structure even after discontinuation (7). Information on the duration of medication use was not available for all participants, which is a limitation of this study; ④ Normal developmental history and physical examination findings (e.g., height and weight); ⑤ No abnormalities on conventional cranial MRI; ⑥ No claustrophobia; ⑦ Good cooperation during the examination; ⑧ Right-handedness.

#### 2.1.2 Exclusion criteria

① Explicit refusal to allow the use of imaging data; ② Poor image quality; ③ Incomplete MRI sequences.

The demographic characteristics of the study participants are summarized in Table 1.

**Table 1:** Demographic characteristics of the study participants by Age group and Sex.

Age group (Years)	Male (*n*)	Female (*n*)	Total (*n*)	Mean age (Years)	Age range (Years)
6–9	128	127	255	7.8 ± 1.1	6–9
10–12	135	135	270	11.2 ± 0.8	10–12
13–15	135	135	270	14.1 ± 0.8	13–15
16–18	127	128	255	17.1 ± 0.7	16–18
Total	495	555	1,100	12.43 ± 3.67	6–18

This study was approved by the Ethics Committee of The Second Affiliated Hospital of Zhejiang Chinese Medical University (Approval No.: ZZYDEL2024113-01). The data used were obtained from previous clinical studies, did not involve sensitive personal patient information, and did not include data or specimens from individuals who explicitly refused their use for research. As no further follow-up or additional information collection from the subjects was required, informed consent was waived.

### 2.2 Methods

Imaging was performed using a United Imaging uMR880 3.0T MRI scanner with a 16-channel head coil. The scanning sequences and parameters were as follows:

#### 2.2.1 T1-weighted imaging (T1WI)

A magnetization-prepared rapid gradient-echo (MP-RAGE) sequence was used with the following parameters: TR 2,250 ms, TE 24 ms, flip angle 8°, acquisition matrix 256 × 256, voxel size 1 mm^3^ isotropic, sagittal slice orientation;

#### 2.2.2 T2-weighted imaging (T2WI)

TR 3,400 ms, TE 110 ms, acquisition matrix 320 × 320, voxel size 0.75 mm × 0.75 mm × 3 mm;

#### 2.2.3 Axial, coronal, and sagittal T2-FLAIR sequences

TR 8,516 ms, TE 166 ms, field of view 240 mm × 240 mm, slice thickness 3 mm, number of excitations 1, acquisition matrix 256 × 256.

#### 2.2.4 Scanning protocol consistency

Throughout the entire data collection period (January 2017–July 2024), all MRI scans were performed on the same United Imaging uMR880 3.0T scanner using the identical 16-channel head coil. A consistent imaging protocol, including the sequences and parameters detailed above, was strictly adhered to for all participants. To ensure data homogeneity and scanner stability, regular quality assurance tests were conducted using a standardized phantom. Furthermore, all acquired images underwent visual inspection for quality control, and any images showing artifacts indicative of scanner drift or performance issues were excluded. This rigorous protocol ensured the consistency and comparability of all imaging data used in this study.

### 2.3 Image post-processing and automated segmentation

**Figure 1:**
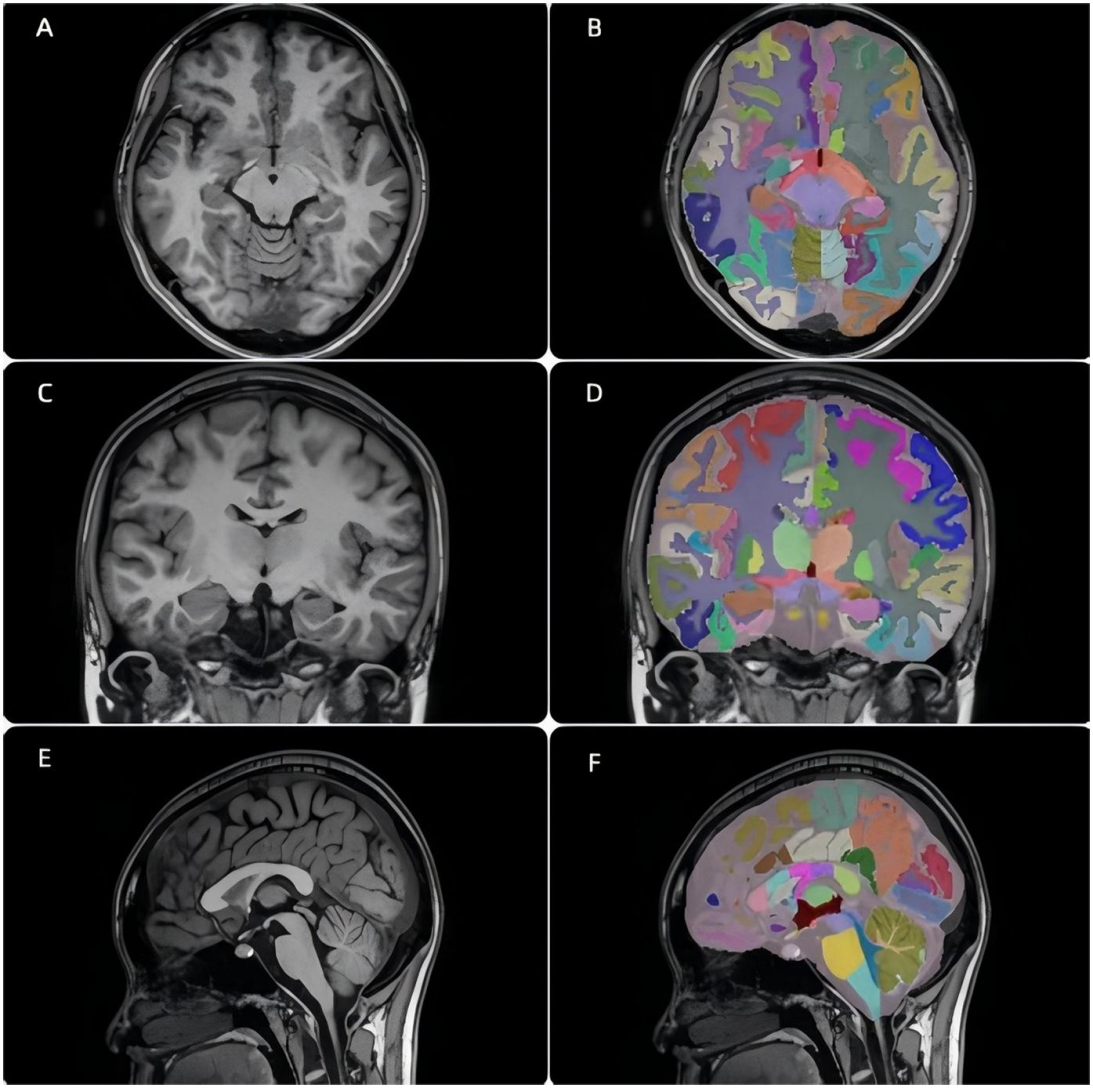
T1-weighted MRI and corresponding AI-based segmentation results in a healthy 10-year-old child. **(A,C,E)**: Axial, coronal, and sagittal T1WI images. **(B,D,F)**: Corresponding segmented brain images.

The axial, coronal, and sagittal T1WI image data from all subjects (Figures 1A,C,E) were first converted to a uniform NIfTI format. Automated whole-brain segmentation was performed using the uAI-Brain Segmentation system (version: v2.1.0), developed by United Imaging Intelligence. The core of this system is a hybrid segmentation algorithm that integrates multi-atlas registration with a deep convolutional neural network based on the 3D U-Net architecture.

#### 2.3.1 Image quality control

Prior to AI segmentation, all T1-weighted images underwent a standardized visual inspection by a single trained radiologist (H.Q., with 3 years of experience in neuroimaging) to assess image quality. Images were excluded if they exhibited significant motion artifacts (e.g., blurring or ghosting), low signal-to-noise ratio (SNR), or poor anatomical clarity. No automated quality control metrics were used; exclusion was based solely on this consistent visual assessment to maintain homogeneity in the input data. The AI segmentation tool required images with minimal motion artifacts and clear gray-white matter contrast for optimal performance.

#### 2.3.2 Segmentation model and training

The deep learning component utilizes a 3D U-Net with five encoding and five decoding levels, connected by skip connections. The model was pre-trained on a large, multi-institutional dataset comprising over 10,000 T1-weighted MRI scans from healthy individuals and patients with various neurological conditions, with manual segmentations reviewed by expert radiologists serving as the ground truth. It should be noted that the pre-training dataset was not specifically enriched with Chinese participants, which could potentially limit its anatomical accuracy for our cohort. To mitigate this limitation and ensure segmentation accuracy, we implemented a multi-faceted strategy: (1) all automated segmentation results underwent rigorous, standardized quality control (QC) and visual inspection by a trained radiologist (as detailed in the Validation and Quality Control section); and (2) during training, data augmentation techniques (including rotation, scaling, and intensity variation) were employed to enhance the model's robustness and generalizability to anatomical variations across different populations. The model was optimized using the Dice loss function and the Adam optimizer.

#### 2.3.3 Segmentation workflow

The specific workflow for each subject's data was as follows:

1. **Preprocessing:** This included N4 bias field correction to address intensity inhomogeneity, non-local means filtering for noise suppression, and intensity normalization to a standard scale.
2. **Registration:** Both affine and nonlinear registration (using the SyN algorithm) were employed to align individual T1-weighted images to the ICBM152 standard template. The ICBM152 template was chosen for consistency with international neuroimaging standards and to facilitate cross-population comparisons. While population-specific templates for Chinese individuals are emerging (8), they were not used here due to limited availability and validation; this may be a limitation for population-specific accuracy.
3. **AI-Based Segmentation:** The preprocessed and registered image was fed into the trained U-Net model. The model outputs a probabilistic label map for over 100 brain regions, including separate labels for left and right hemispheres. The total number of regions of interest (ROIs) generated by the model was 108, encompassing cortical and subcortical structures. The system then automatically identified and extracted the volumes of the following key brain regions for this study: whole brain, gray matter, white matter, cerebral white matter, subcortical gray matter, medial temporal lobe, lateral temporal lobe, occipital lobe, insula, cerebellar gray matter, cerebellar white matter, frontal lobe, parietal lobe, cingulate gyrus, ventricles, cerebrospinal fluid (CSF), and brainstem.
4. **Post-processing:** To refine the raw model output, a standard post-processing pipeline was applied. This included morphological closing operations to fill small holes and connected-component analysis to remove isolated, mis-segmented regions (typically those with a volume of less than 10 voxels).
5. **Volume Calculation:** The system calculated and output both the absolute volume (mL) and the normalized volume (the volume of the structure as a percentage of the total intracranial volume) for each brain region.

#### 2.3.4 Validation and quality control

The segmentation accuracy of the uAI-Brain system was validated prior to this study. The validation was performed against manual segmentations performed by expert radiologists on an internal dataset of 100 T1-weighted scans from both healthy individuals and patients with various neurological conditions. The Dice similarity coefficients (DSC) for major brain regions ranged between 0.85 and 0.95, which is consistent with previously published validation of similar deep learning-based segmentation tools (9). Furthermore, in the present study, all automated segmentation results underwent a standardized quality control (QC) procedure. A trained radiologist (with 3 years of experience in neuroimaging) visually inspected the overlay of segmentation labels on the native T1-weighted images for every subject. Cases with noticeable misalignments or errors (*n* = 15) were excluded from the final analysis, ensuring the robustness of the derived volumetric data.

Final segmented brain images (Figures 1B,D,F) and a brain structure volume report were generated for each subject.

### 2.4 Statistical analysis

Statistical analyses were performed using SPSS software (version 29.0). Normally distributed continuous variables are described as mean ± standard deviation ($\bar{x}\pm s$); non-normally distributed continuous variables are expressed as median (interquartile range). To investigate sex differences in brain structure volumes while controlling for the confounding effect of age, analysis of covariance (ANCOVA) was performed for each brain region, with age included as a continuous covariate. The main effect of sex was examined from this model. Furthermore, to characterize potential differences in developmental trajectories between sexes, a sex-by-age interaction term was added to the ANCOVA model for each region. For brain regions where this interaction was statistically significant (*P* < 0.05 after FDR correction), *post-hoc* analyses were conducted to delineate the sex-specific patterns. The results of the independent samples t-tests (which did not control for age) are superseded by this analysis and are no longer reported. Paired *t*-tests were used to compare volume differences between the left and right sides of brain structures, and Cohen's d was calculated as the effect size for the t-test (i.e., the difference in means divided by the pooled standard deviation). The associations between age and brain structure volumes were assessed using Spearman's rank-order correlation. This non-parametric method was chosen because the normality assumption was violated for many volumetric measures (e.g., normalized volume of the left hippocampus, Shapiro–Wilk W = 0.92, *P* < 0.001). The *P*-values for these correlations were corrected for multiple comparisons using the False Discovery Rate (FDR) method. One-way analysis of variance (ANOVA) was used to compare brain structure volumes among the four age groups. For ANOVA, effect sizes were calculated as partial eta squared (ηp^2^) to quantify the magnitude of differences between groups. For statistically significant inter-group differences, *post-hoc* pairwise comparisons were conducted using the LSD test. Given the multiple statistical tests performed across numerous brain regions, the False Discovery Rate (FDR) method was applied to correct the *P*-values from all t-tests and *post-hoc* ANOVA comparisons. Specifically, we used the Benjamini-Hochberg procedure to control the FDR across all tested brain regions (i.e., all regions were considered as one family of tests). An FDR-corrected *p*-value < 0.05 was considered statistically significant.

## 3 Results

### 3.1 Normalized volumes of intracranial structures in healthy school-aged individuals

The total intracranial volume measured in healthy children and adolescents was (1,517.26 ± 101.02) mL. The normalized volumes (i.e., percentage of total brain volume) of various brain structures are presented in Table 2. The normalized volumes of the third ventricle, fourth ventricle, peripheral cerebrospinal fluid (CSF), and total CSF were 0.09% ± 0.02%, 0.09% ± 0.03%, 17.01% ± 2.10%, and 15.50% ± 2.09%, respectively. The normalized volumes of the left and right sides of the remaining brain regions are detailed in Table 2.

**Table 2:** Normalized volumes of intracranial structures in healthy children and adolescents.

Brain Structure	Normalized volume (%)		Brain Structure	Normalized volume (%)	
	Left	Right		Left	Right
Cerebral White Matter	16.7% ± 0.89%	16.46% ± 0.85%	Medial Orbital Gyrus	0.04% ± 0.06%	0.39% ± 0.06%
Subcortical Gray Matter			Operculum	0.37% ± 0.05%	0.33% ± 0.05%
Caudate Nucleus	0.24% ± 0.22%	0.23% ± 0.03%	Orbital Part	0.22% ± 0.04%	0.84% ± 0.08%
Putamen	0.37% ± 0.04%	0.37% ± 0.04%	Triangular Part	0.32% ± 0.06%	0.37% ± 0.06%
Globus Pallidus	0.24% ± 0.22%	0.24% ± 0.22%	Parietal Lobe		
Thalamus	0.54% ± 0.05%	0.52% ± 0.05%	Postcentral Gyrus	0.72% ± 0.08%	0.70% ± 0.08%
Medial Temporal Lobe			Paracentral Lobule	0.25% ± 0.03%	0.27% ± 0.04%
Hippocampus	0.28% ± 0.05%	0.31% ± 0.06%	Superior Parietal Lobule	0.94% ± 0.12%	0.92% ± 0.11%
Parahippocampal Gyrus	0.13% ± 0.03%	0.12% ± 0.02%	Inferior Parietal Lobule	1.03% ± 0.11%	1.21% ± 0.15%
Amygdala	0.14% ± 0.02%	0.15% ± 0.02%	Precuneus	0.75% ± 0.08%	0.78% ± 0.08%
Entorhinal Cortex	0.11% ± 0.03%	0.12% ± 0.03%	Supramarginal Gyrus	0.99% ± 0.13%	0.87% ± 0.09%
Fusiform Gyrus	0.62% ± 0.07%	0.58% ± 0.07%	Occipital Lobe		
Temporal Pole	0.13% ± 0.04%	0.16% ± 0.05%	Cuneus	0.22% ± 0.04%	0.29% ± 0.05%
Lateral Temporal Lobe			Lingual Gyrus	0.42% ± 0.05%	
Superior Temporal Gyrus	0.92% ± 0.12%	0.89% ± 0.11%	Calcarine Gyrus	0.13% ± 0.03%	0.14% ± 0.04%
Middle Temporal Gyrus	0.78% ± 0.09%	0.86% ± 0.09%	Lateral Occipital Gyrus	0.81% ± 0.09%	0.85% ± 0.12%
Inferior Temporal Gyrus	0.84% ± 0.08%	0.82% ± 0.08%	Cingulate Gyrus		
Planum Temporale	0.16% ± 0.04%	0.18% ± 0.04%	Anterior Cingulate	0.17% ± 0.04%	0.16% ± 0.03%
Heschl's Gyrus	0.09% ± 0.02%	0.07% ± 0.02%	Middle Cingulate	0.12% ± 0.03%	0.16% ± 0.03%
Frontal Lobe			Posterior Cingulate	0.21% ± 0.03%	0.23% ± 0.03%
Precentral Gyrus	0.89% ± 0.09%	0.88% ± 0.09%	lsthmus of Cingulate	0.18% ± 0.02%	0.19% ± 0.02%
Superior Frontal Gyrus	1.62% ± 0.17%	1.54% ± 0.15%	Insula	0.49% ± 0.04%	0.52% ± 0.04%
Rostral Middle Frontal Gyrus	1.45% ± 0.20%	1.54% ± 0.21%	Lateral Ventricle	0.51% ± 0.18%	0.48% ± 0.14%
Caudal Middle Frontal Gyrus	0.41% ± 0.07%	0.42% ± 0.06%	Cerebellum		
Frontal Pole	0.14% ± 0.04%	0.12% ± 0.02%	Cerebellar Gray Matter	3.55% ± 0.30%	3.48% ± 0.34%
Lateral Orbital Gyrus	0.60% ± 0.08%	0.54% ± 0.08%	Cerebellar White Matter	0.93% ± 0.09%	0.86% ± 0.09%

Data are presented as mean ± SD. Volumes are standardized (percentage of total intracranial volume). All structures are labeled with their full names; no abbreviations are used.

### 3.2 Demographic characteristics of the cohort

The demographic characteristics of the study participants are summarized in Table 1. The cohort consisted of 495 males and 555 females, with a balanced distribution across the four age groups [*χ*^2^(3) = 0.00, *P* = 1.000]. The mean age of the entire cohort was 12.43 ± 3.67 years, with no significant differences in age distribution between sexes across the age groups.

### 3.3 Comparison of brain structure volumes between sexes

The analysis of sex differences was performed using ANCOVA with age as a covariate. All reported *P*-values in this section are corrected for multiple comparisons using the False Discovery Rate (FDR) method. This analysis confirmed a significant main effect of sex on several brain structures (Table 3). Males exhibited a larger total intracranial volume (*P* < 0.001, ηp^2^ = 0.032) and a larger normalized volume of the left cerebral white matter (*P* = 0.015, ηp^2^ = 0.005) compared to females. In contrast, females demonstrated larger normalized volumes in the bilateral parahippocampal gyri (left: *P* = 0.023, ηp^2^ = 0.005; right: *P* = 0.011, ηp^2^ = 0.006) and the left fusiform gyrus (*P* = 0.026, ηp^2^ = 0.005).

**Table 3:** Brain structures with a significant main effect of Sex or a significant Sex-by-Age interaction (ANCOVA, controlling for age).

Brain Structure	Main Effect of Sex			Sex-by-Age Interaction	
	*F* value	*P* value	Effect Size (ηp^2^)	*F* value	*P* value
Total Intracranial Volume (mL)	36.15	<0.001^a^	0.032	1.02	0.365
Left Cerebral White Matter	5.95	0.015^a^	0.005	0.45	0.719
Left Parahippocampal Gyrus	5.21	0.023	0.005	0.78	0.508
Right Parahippocampal Gyrus	6.44	0.011	0.006	0.31	0.817
Left Fusiform Gyrus	4.98	0.026	0.005	2.89	0.089
Right Caudate Nucleus	1.25	0.264	0.001	12.50	0.007
Left Rostral Middle Frontal Gyrus	0.88	0.349	0.001	8.95	0.011

a This table presents only brain regions with a significant main effect of sex or a significant sex-by-age interaction after False Discovery Rate (FDR) correction for multiple comparisons (*P* < 0.05). The FDR method controls the expected proportion of false discoveries among all significant findings. Analysis was performed using ANCOVA with age as a covariate. Several regions reported in the initial t-test analysis (e.g., Left Hippocampus, Left Entorhinal Cortex) were no longer significant after adjusting for age and are therefore not listed. Total intracranial volume is presented as the absolute value (mL). Volumes of other structures are normalized (percentage of total intracranial volume). Effect size is reported as partial eta squared (ηp^2^).

Furthermore, to explicitly test for divergent developmental trajectories, sex-by-age interaction terms were included in the model. This analysis revealed significant interactions in the right caudate nucleus (*P* = 0.007) and the left rostral middle frontal gyrus (*P* = 0.011). *post-hoc* analyses indicated that the volume of the right caudate nucleus decreased with age at a significantly faster rate in males than in females. Conversely, a steeper age-related volume reduction in the left rostral middle frontal gyrus was observed in females. No other brain regions showed a significant sex-by-age interaction after FDR correction.

### 3.4 Comparison of left and right normalized volumes of brain structures

Statistically significant differences (FDR-corrected *P* < 0.05) were observed between the left and right normalized volumes in certain regions, including white matter, subcortical gray matter, medial temporal lobe, lateral temporal lobe, frontal lobe, cingulate gyrus, and lateral ventricle. In contrast, no significant differences (FDR-corrected *P* > 0.05) were found between the left and right sides of other brain regions. Detailed statistical results and effect sizes, including exact *P*-values, are presented in Table 4.

**Table 4:** Comparison of left and right normalized volumes of brain structures.^a^

Brain Structure	Left Hemisphere	Right Hemisphere	*t* value	Cohen d	*P* value
Cerebral Gray Matter	23.00% ± 1.03%	23.30% ± 1.10%	−8.64	−1.04	<0.001^a^
Cerebral White Matter	18.47% ± 0.89%	18.21% ± 0.85%	8.25	0.99	<0.001^a^
Subcortical Gray Matter					
Caudate Nucleus	0.24% ± 0.02%	0.23% ± 0.03%	3.62	0.44	<0.001
Thalamus	0.54% ± 0.05%	0.52% ± 0.05%	4.84	0.58	<0.001
Globus Pallidus	0.15% ± 0.02%	0.14% ± 0.02%	2.31	0.28	0.024
Medial Temporal Lobe					
Hippocampus	0.28% ± 0.05%	0.31% ± 0.06%	−5.64	−0.68	<0.001
Parahippocampal Gyrus	0.13% ± 0.03%	0.12% ± 0.02%	4.27	0.53	<0.001
Temporal Pole	0.12% ± 0.04%	0.16% ± 0.05%	−6.36	−0.77	<0.001
Amygdala	0.14% ± 0.02%	0.15% ± 0.02%	−2.02	−0.24	0.048
Entorhinal Cortex	0.11% ± 0.03%	0.12% ± 0.03%	−2.84	−0.34	0.006
Fusiform Gyrus	0.62% ± 0.07%	0.58% ± 0.07%	4.31	0.52	<0.001
Lateral Temporal Lobe					
Superior Temporal Gyrus	0.92% ± 0.12%	0.89% ± 0.11%	2.30	0.28	0.024
Middle Temporal Gyrus	0.78% ± 0.09%	0.86% ± 0.09%	−8.51	−1.03	<0.001
Heschl's Gyrus	0.08% ± 0.02%	0.07% ± 0.02%	4.94	0.59	<0.001
Frontal Lobe					
Superior Frontal Gyrus	1.62% ± 0.17%	1.54% ± 0.15%	5.67	0.68	<0.001
Rostral Middle Frontal Gyrus	1.45% ± 0.20%	1.54% ± 0.21%	−6.45	−0.78	<0.001
Caudal Middle Frontal Gyrus	0.41% ± 0.07%	0.42% ± 0.06%	−2.01	−0.24	0.049
Frontal Pole	0.14% ± 0.04%	0.12% ± 0.03%	3.70	0.03	<0.001
Lateral Orbital Gyrus	0.60% ± 0.08%	0.54% ± 0.08%	8.64	1.04	<0.001
Cingulate Gyrus					
Anterior Cingulate	0.17% ± 0.04%	0.16% ± 0.03%	3.17	0.38	0.002
Middle Cingulate	0.12% ± 0.03%	0.16% ± 0.03%	−9.74	−1.17	<0.001
Lateral Ventricle	0.51% ± 0.18%	0.47% ± 0.14%	2.62	0.31	0.011

Data are presented as mean ± SD. Volumes are standardized (percentage of total intracranial volume).

a indicates statistical significance after False Discovery Rate (FDR) correction for multiple comparisons (*P* < 0.05). The FDR method controls the expected proportion of false discoveries among all significant findings. L, left; R, right.

### 3.5 Differences in normalized volumes of brain structures across Age groups

One-way ANOVA revealed significant differences in normalized volumes across age groups for multiple brain structures (Table 5). The effect sizes (partial eta squared, ηp^2^) for these differences ranged from small to large (e.g., ηp^2^ = 0.12 for right hippocampus to ηp^2^ = 0.42 for left rostral middle frontal gyrus), indicating substantial age-related changes in some regions.

**Table 5:** Normalized volumes of brain structures with significant differences across Age groups.

Brain Structure	One-way ANOVA				Pairwise comparisons *P* value					
	*F* value	Effect Size	*P* value	Adj. P	A&B	A&C	A&D	B&C	B&D	C&D
Peripheral CSF	6.24	0.22	<0.001	<0.001	0.648	0.006	0.019	<0.001	0.003	0.861
Left Cerebral White Matter	3.10	0.13	0.033	0.037	0.049	0.037	0.004	0.938	0.236	0.250
Right Cerebral White Matter	3.82	0.15	0.014	0.035	0.059	0.034	0.001	0.840	0.095	0.124
Left Caudate Nucleus	3.45	0.14	0.021	0.035	0.036	0.012	0.004	0.669	0.277	0.465
Right Hippocampus	2.80	0.12	0.047	0.047	0.613	0.070	0.015	0.154	0.033	0.358
Left Superior Temporal Gyrus	9.70	0.31	<0.001	<0.001	0.660	<0.001	0.003	<0.001	<0.001	0.881
Right Superior Temporal Gyrus	10.32	0.32	<0.001	<0.001	0.037	<0.001	<0.001	0.009	0.005	0.580
Right Middle Temporal Gyrus	3.39	0.14	0.023	0.047	0.541	0.336	0.028	0.085	0.003	0.133
Left Precentral Gyrus	5.49	0.20	0.02	0.035	0.002	<0.001	0.001	0.739	0.674	0.899
Right Planum Temporale	4.49	0.17	0.006	0.035	0.093	0.003	0.002	0.154	0.080	0.613
Left Superior Frontal Gyrus	3.59	0.14	0.018	0.035	0.118	0.003	0.015	0.120	0.275	0.759
Right Superior Frontal Gyrus	3.22	0.13	0.028	0.036	0.022	0.009	0.008	0.768	0.568	0.751
Left Rostral Mid Frontal Gyrus	15.49	0.42	<0.001	<0.001	0.727	<0.001	<0.001	<0.001	<0.001	0.335
Right Rostral Mid Frontal Gyrus	14.30	0.40	<0.001	<0.001	0.695	<0.001	<0.001	<0.001	<0.001	0.344
Left Frontal Pole	6.15	0.22	<0.001	<0.001	0.983	0.006	0.005	0.003	0.002	0.683
Right Frontal Pole	6.73	0.24	<0.001	<0.001	0.149	<0.001	<0.001	0.015	0.002	0.881

Group A: 6–9 years; Group B: 10–12 years; Group C: 13–15 years; Group D: 16–18 years. Volumes are standardized (percentage of total intracranial volume). * indicates statistical significance after False Discovery Rate (FDR) correction for multiple comparisons (*P* < 0.05). The FDR method controls the expected proportion of false discoveries among all significant findings. Effect size is reported as partial eta squared (ηp^2^).

**Figure 2:**
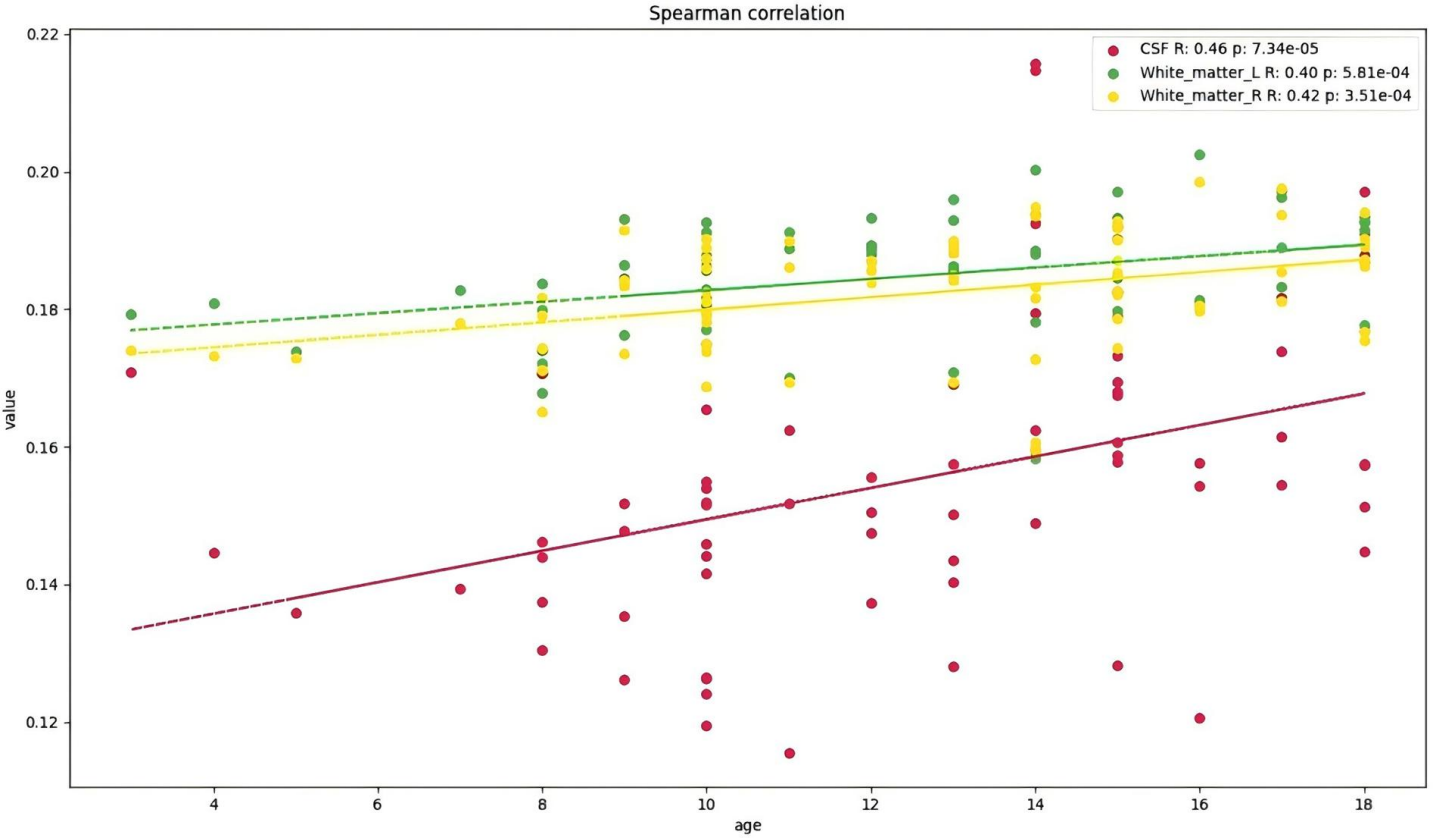
Brain regions showing a positive correlation with age (*p* < 0.05 after FDR correction). *indicates significance after FDR correction. (CSF, Peripheral cerebrospinal fluid; White_matter_L, Left cerebral white matter; White_matter_R, Right cerebral white matter).

Age-related analysis further revealed two distinct developmental trajectories: On one hand, the volumes of CSF spaces (e.g., peripheral CSF) and bilateral white matter expanded with age (positive correlation, e.g., peripheral CSF: *P*-FDR = 0.0001) (Figure 2). On the other hand, multiple gray matter regions (particularly in the frontal lobe, temporal lobe, and some subcortical nuclei) generally exhibited an age-related pruning or reduction trend (negative correlation, e.g., left precentral gyrus: *P*-FDR = 0.013; right hippocampus: *P*-FDR = 0.028).

**Figure 3:**
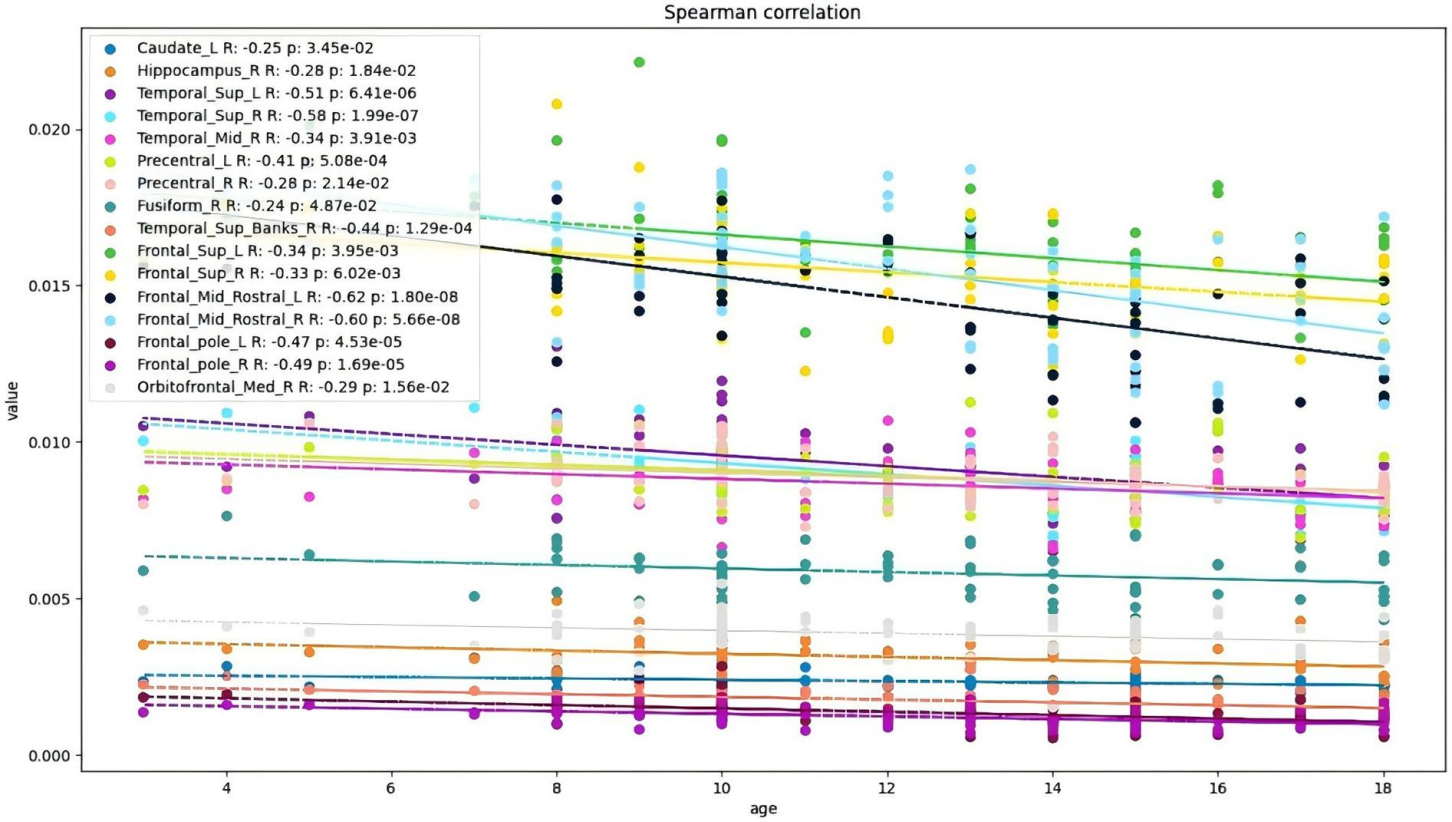
Brain regions showing a negative correlation with age (*P* < 0.05 after FDR correction). *indicates significance after FDR correction.

Specifically, positive correlations were observed in peripheral CSF and bilateral white matter (FDR-corrected *P* < 0.001), while negative correlations were widespread in the frontal lobe (e.g., bilateral precentral gyrus, superior frontal gyrus, anterior middle frontal gyrus, frontal pole, etc.; FDR-corrected *P* < 0.05) (Figure 3), temporal lobe (e.g., bilateral superior temporal gyrus, right middle temporal gyrus, etc.; FDR-corrected *P* < 0.05), as well as medial temporal lobe and subcortical structures (e.g., right hippocampus, fusiform gyrus, and left caudate nucleus; FDR-corrected *P* < 0.05).

## 4 Discussion

Leveraging deep learning-based automated brain segmentation, this study provides the first systematic report on standardized reference values for brain structural volumes in healthy Chinese children and adolescents aged 6–18 years. The main findings include: (1) Significant sexual dimorphism and laterality in brain volumes: Males exhibited larger total intracranial volume and left cerebral white matter volume, while Females showed larger volumes in the bilateral parahippocampal gyri and left fusiform gyrus. In addition, widespread hemispheric asymmetry was observed in white matter, subcortical gray matter, and multiple lobar regions. (2) Heterogeneous developmental patterns of different brain tissue components with age: cerebrospinal fluid (CSF) spaces and white matter volume were positively correlated with age, while multiple gray matter regions (e.g., frontal lobe, temporal lobe, and some subcortical nuclei) exhibited an age-related reduction. These findings illustrate the dynamic structural remodeling of the brain during childhood and adolescence. The observed sexual dimorphism, particularly in limbic and temporal regions, invites consideration of underlying biological mechanisms. The larger total intracranial volume in males is a well-replicated finding, which has been hypothesized to relate to pubertal sex hormones such as testosterone (10). Conversely, the relatively larger normalized volumes of the bilateral parahippocampal gyri and left fusiform gyrus in females may reflect neurodevelopmental trajectories influenced by estrogen. Estrogen has been shown to exert neurotrophic and protective effects on cortical and hippocampal structures, potentially modulating synaptic density and gray matter volume in regions critical for memory and emotional processing (11, 12). While not measured in this study, Genetic factors also likely contribute to these population-specific patterns. For instance, alleles in genes related to brain-derived neurotrophic factor (BDNF) or sex hormone receptors, which exhibit varying frequencies across ethnic populations, could interact with hormonal milieus to shape distinct regional volumetric patterns during development (13). While our cross-sectional data cannot definitively establish causality, the confluence of hormonal influences during the adrenarche and pubertal stages covered by our age range (6–18 years) provides a plausible framework for interpreting these sex-specific developmental trajectories. Future longitudinal studies integrating hormone assays and genetic data are essential to directly test these hypotheses within the Chinese pediatric population. Beyond sex and age, ethnocultural factors—encompassing genetic ancestry, dietary patterns, socioeconomic status, and educational practices—likely contribute to the population-specific differences we observed. For instance, genetic polymorphisms with differing allele frequencies across populations, such as the BDNF rs6265 Met allele which is more prevalent in Asian populations, have been linked to variations in hippocampal and prefrontal cortex volume (14). This could provide a plausible biological mechanism for the volumetric differences we noted in medial temporal lobe structures compared to Western cohorts. Beyond genetics, environmental and cultural exposures may also sculpt the developing brain. Dietary patterns rich in omega-3 fatty acids, common in certain Chinese culinary traditions, could support white matter integrity, potentially contributing to the observed developmental trends. Furthermore, intensive educational practices in China that emphasize mathematics and language acquisition from an early age might drive experience-dependent structural adaptations in regions critical for executive function and visuospatial processing, such as the frontal lobes and intraparietal sulcus (15). The confluence of these genetic and culturally-mediated environmental factors offers a more substantive framework for interpreting the unique developmental trajectories identified in our study. For instance, the significant age-related volume reductions we observed in frontal lobe regions, particularly in the rostral middle frontal gyrus, may be partly shaped by culturally-specific experiences such as China's intensive educational practices. The rostral middle frontal gyrus is a key node for higher-order executive functions like cognitive control, working memory, and problem-solving. The Chinese educational system emphasizes sustained engagement in these very cognitive domains from an early age, especially in mathematics and language acquisition. We hypothesize that this sustained, high-demand cognitive training drives experience-dependent plasticity, accelerating the neural specialization and efficient synaptic pruning within the prefrontal cortex (16, 17), potentially manifesting as the steeper age-related volumetric decline we documented in these frontal regions compared to Western cohorts where educational pressures may differ. These educational influences may provide a plausible explanation for the specific volumetric patterns we observed. In our results, the rostral middle frontal gyrus showed one of the largest age-related volume reductions (ηp^2^ = 0.42, see Table 5), with steeper declines in females as indicated by the significant sex-by-age interaction (*P* = 0.011, see Table 3). This region's pronounced developmental trajectory may reflect the intensive cognitive training characteristic of Chinese education, which could drive accelerated synaptic pruning and neural efficiency in prefrontal circuits supporting executive functions. Similarly, the widespread frontal lobe volume reductions we documented (including superior frontal gyrus, frontal pole, and precentral gyrus) align with the cognitive domains most heavily engaged by China's academically rigorous curriculum. This underscores the necessity of considering ethnocultural context, including specific learning experiences, when interpreting developmental brain changes. The confluence of these genetic and culturally-mediated environmental factors offers a more substantive framework for interpreting the unique developmental trajectories identified in our study. While the present study was not designed to dissect these specific contributions, the volumetric differences we identified provide a compelling rationale for future research that directly integrates genetic, dietary, and socio-cultural metrics to elucidate the mechanisms underlying ethnocultural variation in brain development. Future longitudinal studies should incorporate genetic profiling (e.g., BDNF, COMT), hormonal assays, and detailed socio-cultural metrics (e.g., parental education, dietary logs) to directly test how these factors interact to shape brain development in Chinese children. This endeavor moves beyond mere normative data reporting; it serves as a critical test of the generalizability of neurodevelopmental models. The population-specific reference standards we established provide a foundational tool for precision medicine by enabling future clinicians and researchers to: 1) objectively quantify individual brain development against a Chinese-specific norm, and 2) establish whether structural alterations observed in disorders like childhood-onset schizophrenia or autism spectrum disorder in Chinese patients represent universal pathophysiological mechanisms or population-specific manifestations.

The brain volumetric reference standards established in this study for Chinese children and adolescents can be directly contextualized within the growing body of literature employing AI-based segmentation. As we set out to investigate in the Introduction, our findings demonstrate that the well-established neurodevelopmental trajectories from Western cohorts are not universally applicable to Chinese youth, revealing population-specific variations in both absolute volumes and developmental patterns. The accuracy and efficiency of our U-Net-based approach are supported by studies demonstrating the high reliability of similar deep learning architectures, such as the FastSurfer pipeline, for volumetric assessment (18). Furthermore, our observation of age-related white matter expansion and gray matter pruning aligns with the developmental trajectories captured in large-scale imaging cohorts. Notably, while overarching neurodevelopmental patterns (e.g., age-related gray matter pruning and white matter expansion) are largely consistent with those reported in large Western cohorts (19), direct comparison of our volumetric values reveals population-level differences that substantiate the concern of potential misclassification. For instance, the total intracranial volume (TIV) in our Chinese cohort (1,517.26 ± 101.02 mL) appears systematically different from averages reported in predominantly European samples of similar age ranges, such as those from the UK Biobank study (20). To provide a concrete example, the mean normalized volume of the left hippocampus in our cohort was 0.28% of TIV (Table 2). In contrast, large-scale studies of comparable Western pediatric populations, such as the Pediatric Imaging, Neurocognition, and Genetics (PING) Study, have reported mean normalized hippocampal volumes of approximately 0.33% of TIV (21, 22). This represents a relative difference of over 15%. If the Western norm of 0.33% were applied as a clinical cutoff, a significant proportion of children in our study with biologically normal, smaller hippocampi (e.g., those at the 30th percentile of our distribution) might be flagged as potentially abnormal. This illustrates how the application of non-population-specific standards can introduce systematic bias and increase the risk of false positives in clinical practice and research. While our findings suggest that population-specific norms could theoretically reduce such misclassification, direct evidence from clinical cohorts is needed to confirm this benefit. Similarly, for the frontal lobe, our data show a normalized volume of the left superior frontal gyrus at 1.62% of TIV, which differs from the value of approximately 1.75% reported in studies of the Philadelphia Neurodevelopmental Cohort (23, 24), further underscoring the need for population-specific benchmarks. Furthermore, the specific patterns of sexual dimorphism we identified—such as significantly larger normalized volumes of the bilateral parahippocampal gyri and left fusiform gyrus in females—are either less pronounced or exhibit a different regional emphasis compared to Western norms (25). These discrepancies are not merely statistical but carry practical implications. Applying Western-derived reference percentiles for structures like the hippocampus or frontal lobe volumes to our cohort could potentially misclassify a portion of healthy Chinese children as outliers, thereby obscuring true pathological findings or generating false positives. While our findings suggest a theoretical advantage of population-specific standards in reducing misclassification, it is important to note that this study does not provide direct empirical evidence for this claim. Future studies involving clinical cohorts are needed to validate whether these reference values actually improve diagnostic accuracy. This direct, albeit preliminary, comparison strengthens the conclusion that population-specific standards are not merely preferable but necessary for accurate assessment in clinical and research settings for Chinese youth. even when employing the same advanced segmentation technology, applying standards derived from one population may lead to misinterpretation of individual brain development in another.

Multiple factors, including sex, age, genetic background, and cultural environment, are significantly associated with brain structural development during school age (26, 27). In our statistical models, we controlled for age as a continuous covariate in all ANCOVAs examining sex differences, and we also tested for sex-by-age interactions to capture potential divergent developmental trajectories. However, due to the cross-sectional nature of our study and the lack of detailed genetic or socio-cultural metrics in our dataset, we were unable to incorporate these additional covariates. Future longitudinal studies incorporating genetic and environmental data are warranted to further disentangle their contributions.

The automated brain segmentation technique employed in this study is based on a multi-atlas registration algorithm. The core mechanism involves large-deformation diffeomorphic metric mapping (LDDMM) to nonlinearly align the target image with pre-segmented reference templates. The registration process utilized the ICBM152 standard brain template released by the International Consortium for Brain Mapping (ICBM), supported by the FMRIB Software Library (FSL) developed by the University of Oxford (28). By establishing an inverse mathematical transformation model, segmentation results from the template space were accurately mapped back to the original image space, achieving high-precision brain parcellation.

Normal brain tissue primarily consists of gray matter, white matter, and cerebrospinal fluid. Among MRI sequences, T1-weighted imaging (T1WI) offers superior tissue contrast compared to T2-weighted imaging (T2WI) and fluid-attenuated inversion recovery (FLAIR). Therefore, T1WI was used for conventional brain segmentation in this study (29). In this study, we utilized T1-weighted MRI for automated segmentation due to its superior tissue contrast. Future studies could integrate multimodal imaging (e.g., T2WI, FLAIR) to extend these reference standards to pathological conditions such as white matter lesions.

Our analysis, which controlled for age as a covariate, revealed distinct patterns of sexual dimorphism. Specifically, males exhibited a larger total intracranial volume, whereas females showed larger normalized volumes in the bilateral parahippocampal gyri and left fusiform gyrus. The finding of larger total intracranial volume in males is a robust and consistently reported neurodevelopmental pattern across diverse populations, including Chinese cohorts (30). The larger normalized volumes in the limbic and temporal regions (parahippocampal and fusiform gyri) among females, while less commonly a focus in Western studies, resonate with emerging evidence suggesting unique patterns of sexual dimorphism in the developing Asian brain (4). The establishment of these population-specific reference data is critical, as the application of normative standards derived from other populations might fail to detect or misinterpret such nuanced differences. The finding of larger total intracranial volume in males is a robust and consistently reported neurodevelopmental pattern across diverse populations (31), and has been specifically replicated in studies of Chinese pediatric and adolescent cohorts (32). The larger normalized volumes in the limbic and temporal regions (parahippocampal and fusiform gyri) among females, while less commonly a focus in Western studies, resonate with emerging evidence from Asian populations, including studies of young Chinese adults, suggesting unique patterns of sexual dimorphism in the developing Asian brain (30, 33). The biological basis for these specific regional differences, however, remains unclear and warrants further investigation.

Neuroimaging studies have confirmed that lateralization of brain structural volumes is a common feature of the typical human brain across diverse populations (34). The significant hemispheric asymmetries we observed in regions such as white matter, the caudate nucleus, thalamus, and multiple frontal and temporal gyri (Table 4) are consistent with the well-documented structural lateralization of the human brain (35). Furthermore, the detailed maps of hemispheric asymmetry established in this study may have clinical relevance for diagnosing neurodevelopmental disorders. For instance, autism spectrum disorder (ASD) is frequently characterized by aberrant patterns of brain lateralization, including reduced or reversed hemispheric asymmetries in language-related regions such as the planum temporale and inferior frontal gyrus, as well as in social cognition networks (36). The population-specific normative ranges for structural asymmetry provided by our study could serve as a benchmark to identify such deviations in Chinese children with ASD. By comparing individual asymmetry patterns against these reference standards, clinicians might improve the detection of atypical neurodevelopment. Similarly, other neurodevelopmental conditions such as childhood-onset schizophrenia have also been associated with altered structural lateralization (37). Our data thus provide a foundation for future research exploring the diagnostic and prognostic value of population-specific asymmetry measures in neurodevelopmental disorders. Our study provides quantitative, population-specific reference data for structural asymmetries in Chinese youth. The significant hemispheric asymmetries we observed provide a quantitative, population-specific baseline for structural lateralization in Chinese youth. Our study provides quantitative, population-specific reference data for structural asymmetries in Chinese youth.

All participants in this study were right-handed healthy children and adolescents. In this population, the left hemisphere exhibited larger volumes in white matter, caudate nucleus, thalamus, globus pallidus, parahippocampal gyrus, fusiform gyrus, superior temporal gyrus, transverse temporal gyrus, superior frontal gyrus, frontal pole, lateral orbitofrontal cortex, anterior cingulate cortex, and lateral ventricle (38). In contrast, the right hemisphere showed larger volumes in gray matter, hippocampus, temporal pole, amygdala, entorhinal cortex, middle temporal gyrus, anterior and posterior middle frontal gyrus, and middle cingulate cortex. These findings demonstrate significant hemispheric asymmetries in brain structure, which are consistent with previous reports of structural lateralization in the human brain (39). The population-specific reference standards for structural asymmetry established here provide a quantitative baseline that is essential for identifying pathological deviations. By defining the normal range of asymmetry in the developing Chinese brain, our data enable a more precise exploration of neurobiological markers for psychiatric disorders known to involve aberrant lateralization (40).

Our results are consistent with established neurodevelopmental trajectories, where cortical volume changes typically follow a posterior-to-anterior sequence, with widespread increases in childhood and selective reductions during adolescence (41). This pattern of progressive gray matter pruning and white matter consolidation is thought to be, in part, orchestrated by hormonal changes during puberty, which refine neural circuits by promoting synaptic elimination and enhancing axonal insulation (42). Specifically, we observed age-related volume reductions in frontal and temporal regions, supporting this pattern. In this study, standardized volumes of peripheral CSF and bilateral white matter differed across age groups and were positively correlated with age. In contrast, standardized volumes in the bilateral precentral gyrus, bilateral superior frontal gyrus, bilateral anterior middle frontal gyrus, bilateral frontal pole, right medial orbitofrontal cortex, bilateral superior temporal gyrus, right middle temporal gyrus, right planum temporale, right hippocampus, right fusiform gyrus, and left caudate nucleus also showed differences but were negatively correlated with age. These results suggest that developmental patterns of brain structural volumes vary across regions in healthy school-age individuals, yet generally follow a linear trend with age. Some brain regions become more developed with age, which is largely consistent with previous research, though discrepancies exist. For instance, some studies conducted in Western cohorts have reported a negative correlation between white matter and peripheral CSF volume and age in school-age children (43, 44), which contradicts the positive correlation observed in our Chinese cohort. Such discrepancies may arise from several factors: first, differences in the age range of study populations may lead to heterogeneous developmental trajectories; second, variations in MRI scanning parameters (e.g., field strength, sequence, resolution) may affect volume measurement accuracy; furthermore, differences in automated segmentation algorithms and their training datasets could also contribute to inconsistent results. Despite these methodological variations, this study—based on a relatively large sample size (*n* = 1,150) and more refined age grouping (4 groups)—yields representative and robust results, providing a reliable foundation for establishing brain development reference values for Chinese children and adolescents.

Future longitudinal studies that integrate genetic information, detailed hormonal assessments, and these volumetric data are warranted to elucidate the biological mechanisms—including the specific roles of pubertal hormones and genetic polymorphisms—underlying the developmental trajectories and sexual dimorphism we observed.

The population-specific reference standards established herein provide a normative baseline that could be used in future clinical research to investigate whether they improve diagnostic accuracy for neurodevelopmental disorders. For instance, in future diagnostic settings, these reference standards could potentially allow radiologists and clinicians to objectively quantify individual brain development against a population-specific norm, which might reduce the dependence on subjective visual assessment and standards derived from other populations. The identified sexual dimorphism in limbic structures, established here in a healthy cohort, provides population-specific normative data that may serve as a baseline for future studies investigating sex differences in neuropsychiatric disorders. Furthermore, based on the age-related volumetric changes we documented, the detailed trajectories provide a baseline that could be used in future clinical studies to detect accelerated or delayed maturation in children at risk for neurodevelopmental disorders. This volumetric reference framework with genetics and multimodal neuroimaging in future studies could help identify sensitive imaging-genetic markers, enhancing our understanding of normal and aberrant brain development.

Furthermore, the detailed maps of hemispheric asymmetry established in this study provide a population-specific quantitative baseline for typical brain development, which is essential for research into neuropsychiatric disorders where aberrant structural lateralization has been implicated (45, 46). Our study provides a structural reference that could facilitate more accurate identification and interpretation of pathological deviations in future comparative studies of Chinese populations (47, 48). Our findings thus pave the way for future studies aiming to elucidate the ethnocultural and genetic determinants of brain maturation by providing a necessary phenotypic reference for large-scale imaging-genetics studies in Chinese populations. Furthermore, the structural references we established allow pathological deviations in disorders like schizophrenia to be more accurately identified and interpreted by providing a population-specific benchmark that minimizes confounding from normal ethnic variation.

This study has several limitations that warrant consideration. First, its cross-sectional nature captures differences between age groups but cannot track dynamic developmental trajectories within individuals. Consequently, while we observe patterns of age-related volume change (e.g., gray matter reduction), these data cannot clarify whether these changes represent continuous maturational processes or potential cohort effects (e.g., generational differences in nutrition or lifestyle). Second, although our sample size (*n* = 1,100) is substantial, the data originate from a single center. This limits the generalizability of our reference standards to the broader Chinese pediatric population, which encompasses considerable genetic and environmental diversity. Future multi-center studies with harmonized imaging protocols are essential to establish nationally representative norms. Third, we did not collect or analyze detailed metrics of socioeconomic status (SES), such as parental education, occupation, or family income, from our participants. SES is a well-established determinant of brain structure and cognitive development, influencing factors such as nutrition, cognitive stimulation, and stress levels (49, 50). Although formal SES metrics were not collected, our sample was recruited from a single urban center in Hangzhou, which may over-represent children from families with relatively higher educational and income levels compared to the broader Chinese population that includes vast rural areas. Consequently, the generalizability of our reference values to children from rural areas or from families with lower SES may be limited. Future studies aiming to establish population-wide norms should incorporate comprehensive SES assessments to better account for this important source of variation. Fourth,the exclusion of left-handed and mixed-handed individuals introduces a selection bias. Given that handedness is linked to cerebral lateralization, our cohort—comprising exclusively right-handed participants—may not fully represent the structural variations present in the general population. This limits the applicability of our reference values to non-right-handed children and adolescents. Future studies should include these groups to provide a more comprehensive reference database. Finally, our study did not include cognitive or behavioral assessments; therefore, the functional implications of the observed structural differences remain unknown and represent an important direction for future research. Despite these limitations, the robust reference values provided here form an essential foundation for designing and interpreting such future longitudinal and multi-center studies.

In conclusion, by applying automated brain segmentation, this study establishes the first set of population-specific reference standards for brain structural volumes in Chinese children and adolescents. Our data reveal distinct developmental trajectories and volumetric norms compared to Western cohorts, underscoring that the application of non-population-specific standards could lead to misclassification. These findings provide a critical, culturally relevant structural reference for monitoring typical and atypical brain development in the Chinese pediatric population.cents, establishing a critical structural reference for monitoring typical brain development in the Chinese pediatric population.

## Data Availability

The original contributions presented in the study are included in the article/Supplementary Material, further inquiries can be directed to the corresponding authors.
